# Testicular shear wave elastography in oligo-astheno-teratozoospermic individuals: a prospective case–control study

**DOI:** 10.1007/s11255-021-02909-4

**Published:** 2021-06-10

**Authors:** Ester Illiano, Francesco Trama, Antonio Ruffo, Giuseppe Romeo, Filippo Riccardo, Felice Crocetto, Fabrizio Iacono, Elisabetta Costantini

**Affiliations:** 1 Andrology and Urogynecology Clinic, Santa Maria Terni Hospital, University of Perugia, Viale Tristano di Jannuccio 1 Terni, Perugia, Italy; 2 Andrea Grimaldi Hospital, San Giorgio a Cremano (NA), Naples, Italy; 3 Urology Department, A.O.R.N. A. Cardarelli, Naples, Italy; 4 Department of General and Specialized Surgeries, Renal Transplantation, Nephrology, Intensive Care and Pain Management, University of Federico II, Naples, Italy

**Keywords:** Shear wave elastography, Oligo-astheno-teratozoospermia, Male infertility, Testicular stiffness

## Abstract

**Purpose:**

The primary objective of this study was to evaluate the testicular stiffness by ultrasound shear wave elastography (SWE) both in men with oligo-astheno-teratozospermia (OAT) and in control group. The secondary objective was to identify a possible correlation between semen quality with testicular stiffness.

**Methods:**

This was a prospective case-control study. We divided the sample in two groups; Group A (case group) included men with OAT, and Group B (control group) men with normal sperm parameters. All participants had at last two semen analysis in the past 180 days (at last 90 days apart), using performed ultrasound and SWE elastography.

**Results:**

We analyzed 100 participants, 50 patients in Group A and 50 controls in Group B. There were statistically significant differences in term of testicular volume and testicular stiffness between two groups. Men with OAT had the testicular stiffness value higher than the controls in both sides (left testicular stiffness 21.4 ± 5.4 kPa vs 9.9 ± 1.6 kPa, *p* < 0.0001; right testicular stiffness 22.9 ± 4.8 kPa vs 9.5 ± 2.4 kPa, *p* < 0.0001). Men with abnormal semen parameters showed an inverse correlation between the mean value of testicular stiffness and total sperm count (22.15 ± 3.38 kPa, *r* = − 0.387, *p* = 0.005), sperm concentration (22.15 ± 3.38 kPa, *r* = − 0.244, *p* = 0.04), and progressive motility (22.15 ± 3.38 kPa, *r* = − 0.336, *p* = 0.01), while the correlation was not evident in controls group.

**Conclusion:**

SWE is able to differentiate between testicles with spermatogenic changes from a healthy testicle. For this reason, it could be used to evaluate, in a non-invasive way, the tissue alterations of the organ.

## Introduction

Infertility is estimated to affect between 8 and 12% of reproductive-aged couples worldwide [1]. Approximately 40% of all male infertility cases are mainly caused by sperm defects [2]. Oligo-astheno-teratozoospermia syndrome (OAT) is defined as the presence of oligozoospermia (< 15 million spermatozoa/mL), asthenozoospermia (< 32% progressive motile spermatozoa), and teratozoospermia (< 4% normal forms).

In the diagnostic work-up of OAT syndrome, B-mode ultrasonography is used specifically in the measurement of testicular volume and echogenicity, and in the detection of possible varicocele, seminal vesicle abnormalities, and abnormalities in the conformation of the epididymis [3].

Ultrasound (US) shear wave elastography (SWE) has been widely used in the past few years in the andrological field. It is an imaging modality that allows the evaluation of tissue stiffness based on the trajectory of shear wave propagation through a structure [4]. SWE, in which an ultrasonic pulse is applied to the tissue in SWE, induces the formation of transverse waves arranged perpendicularly to the direction of the ultrasound beam [5]. Higher shear wave velocities correspond to harder tissue. Using SWE, a quantitative measurement of the stiffness of a tissue is obtained by calculating the modulus of elasticity (or Young's modulus) expressed in Kilopascal (kPa) or meter per second (m/s) [5].

SWE is used to investigate testicular pathologies, in particular neoplastic processes [6], infarction [7], torsion [8], varicocele [9], and orchitis [10]. There are only a few previous studies in the literature that have investigated the relationship between SWE values and defects of semen quality [11, 12].

The primary aim of our study was to analyze testicular stiffness in both the control and OAT groups. The secondary aim of this study was to correlate testicular stiffness with testicular volume and to identify a possible correlation of SWE values with semen quantity and quality.

## Materials and methods

This study was a prospective case–control study. The study was approved by the local ethics committee (CER 3767/20) and each participant included in the study signed an informed consent to participate in the study. The study was conducted in accordance with the Privacy Act and in accordance with the Declaration of Helsinki in all applicable aspects.

We divided the participants into two groups: Group A (case group) included males aged > 18 years with abnormal semen parameters; in particular, all participants had OAT syndrome according to the WHO 2010 criteria [13].

Group B (control group) included healthy male participants > 18 years of age who underwent testicular ultrasound for unrelated reasons and who had no alteration of seminal parameters, or no testicular affections.

Exclusion criteria were obstructive azoospermia (OA) and non-obstructive azoospermia (NOA), presence of monorchid, varicocele, hydrocele, urogenital infections, history of testicular tumors or twisting of the funiculus, history of cryptorchidism, previous genital surgery in the past 12 months, and Klinefelter syndrome.

In both the groups, we gathered a detailed clinical history and performed local examination, US, and SWE elastography. All SWE procedures were performed by a single urologist (who had 3 years of experience in SWE elastography) in a dedicated room. Another senior urologist with 5 years of experience in SWE elastography verified the data in the picture archive and communications system (PACS) to ensure that the measurements were accurate. The ultrasound scanner with the integrated SWE module [General Electric (GE) Logiq S8, Chicago, USA] and a linear probe (7.5–13.5 MHz) was used.

**Fig. 1:**
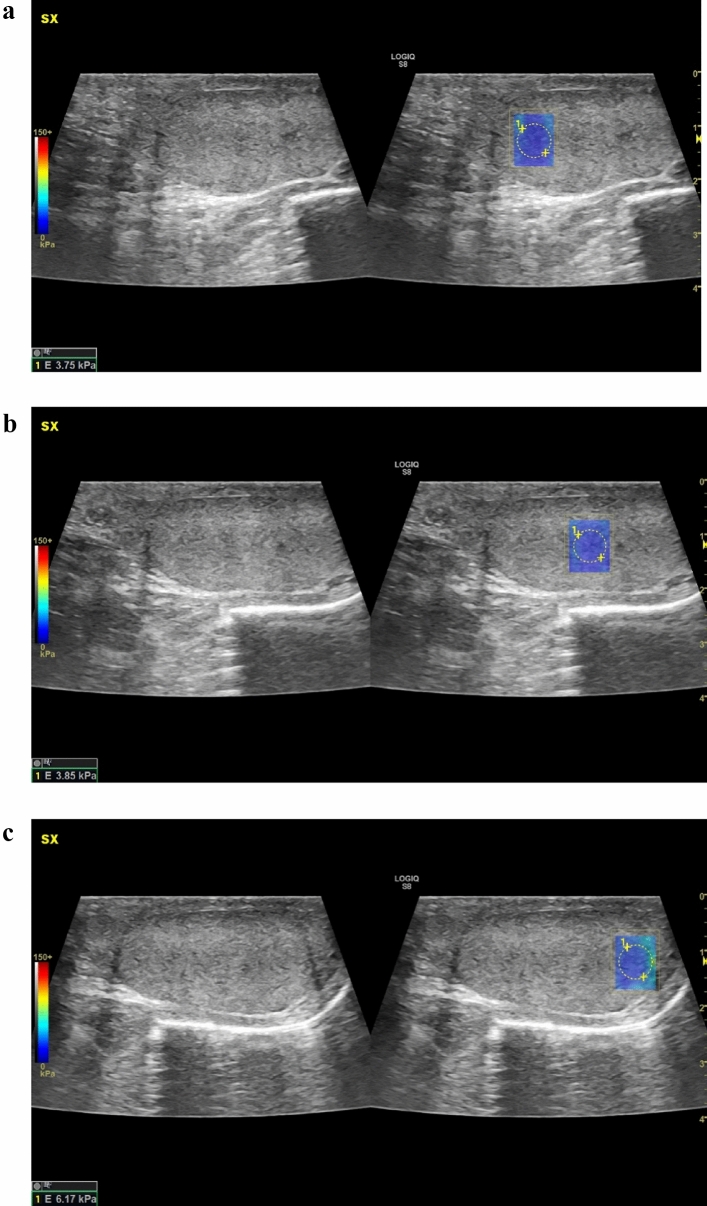
Testicle was divided into three parts: 1/3 upper pole (**a**), 1/3 central region (**b**), 1/3 lower pole (**c**) and the measurement was performed in the axial plane by affixing a region of interest (ROI). Each ROI provided a mean shear wave stiffness value in kPa

In mode B, the testicular volumes were measured by applying the ellipsoid formula [length × height × width × 0.523]. Then, SWE was performed. The stiffness values were calculated separately for the two testicles. Each testicle was divided into three parts (Fig. 1): 1/3 upper pole (Fig. 1a), 1/3 central region (Fig. 1b), and 1/3 lower pole (Fig. 1c). The measurement was performed in the axial plane by affixing a region of interest (ROI). Each ROI provided a mean shear wave stiffness value in kPa. We then calculated the average of the three regions examined. The transducer was touched laterally to minimize operator-dependent pressure on the testicular region, and an ample coupling gel was used. In addition to the value calculated in m/s and in kPa, it was possible to see a color scale according to the stiffness: intense blue color indicates elasticity, while a deep red color indicates stiffness. Each patient was asked to hold their breath when measuring stiffness in order to reduce movement artifacts.

All participants had at least two semen analysis performed in the last 180 days (at least 90 days apart) at the same diagnostic center that complied with the WHO 2010 criteria.

Furthermore, the recommended abstinence period was 2–7 days as recommended by the 2010 WHO guidelines.

Statistical analysis was performed using the paired t-test for continuous parametric variables and the Mann–Whitney test and Wilcoxon's test for nonparametric variables. The Kolmogorov–Smirnov test was used to evaluate whether the data were normally distributed and Pearson’s correlation test was used for quantitative variables. All calculations were performed using the IBM-SPSS^®^ version 22.0 (IBM Corp., Armonk, NY, USA). We considered *p* < 0.05 to indicate statistical significance.

## Results

A total of 114 participants were declared eligible for the study from June 2019 to November 2020. Eleven participants did not wish to perform the proposed examination and for three participants, the measurements were not reliable due to movement artifacts. A total of 100 participants were finally enrolled: 50 patients in Group A, and 50 controls in Group B.

Table 1 shows the demographic data of both the groups. The male participants in the two groups were comparable by age and BMI (*p* > 0.05). Moreover, there were no statistically significant differences between the semen parameters in both groups between the first determination and the second determination made.

**Table 1:** Average age and BMI of male patients and their partners

	Male mean age (years) ± SD	*p* value	BMI (kg/m^2^) ± SD	*p* value	Female mean age (years) ± SD	*p* value
GROUP A	36 ± 16.7	0.970	25.2 ± 6.2	0.884	30 ± 8	0.942
GROUP B	38 ± 17.4		25.6 ± 5.8		30 ± 8.2	

*SD* standard deviation

*Statistically significant at *p* < 0.05

Table 2 shows the seminal fluid values in groups A and B. There was a statistically significant difference (*p* < 0.0001) between group A and group B in terms of total sperm count (million/ejaculate), sperm concentration (million/mL), progressive motility (%), and normal forms (%).

**Table 2:** Semen parameters in group A and in Group B

Parameters	Group A	Group B	*p* value
Normal viscosity, no pts	35	38	nd
Abstinence days	3.8 ± 1.6	3.9 ± 1.7	nd
Complete fluidification, no pts	34	36	nd
PH (mean ± SD) [r.v.: pH > 7.2]	8.1 ± 0.1	8.4 ± 0.3	0.219
Semen volume (mL, mean ± SD) [r.v.: semen volume: > 1.5 mL]	2.5 ± 1.2	2.9 ± 0.9	0.115
Total sperm number (10^6^/ejaculate, mean ± SD) [r.v. total sperm number > 39 × 10^6^/ejaculate]	25 ± 9.6	69.1 ± 21.2	< 0.0001*
Sperm concentration (10^6^/mL, mean ± SD) [r.v. sperm concentration > 15 × 10^6^/mL]	7.7 ± 4.2	26.2 ± 7.7	< 0.0001*
Progressive motility (mean ± SD) [r.v.: PR > 32%]	18.4 ± 9.2	48.9 ± 10.9	< 0.0001*
Sperm morphology (normal forms %) [r.v. > 4%]	2.5 ± 1.1	15.8 ± 7.1	< 0.0001*

*PR* progressive motility, *Pts* patients, *R.V.* reference value, *nd* not determined

*p ≤ 0.05

**Fig. 2:**
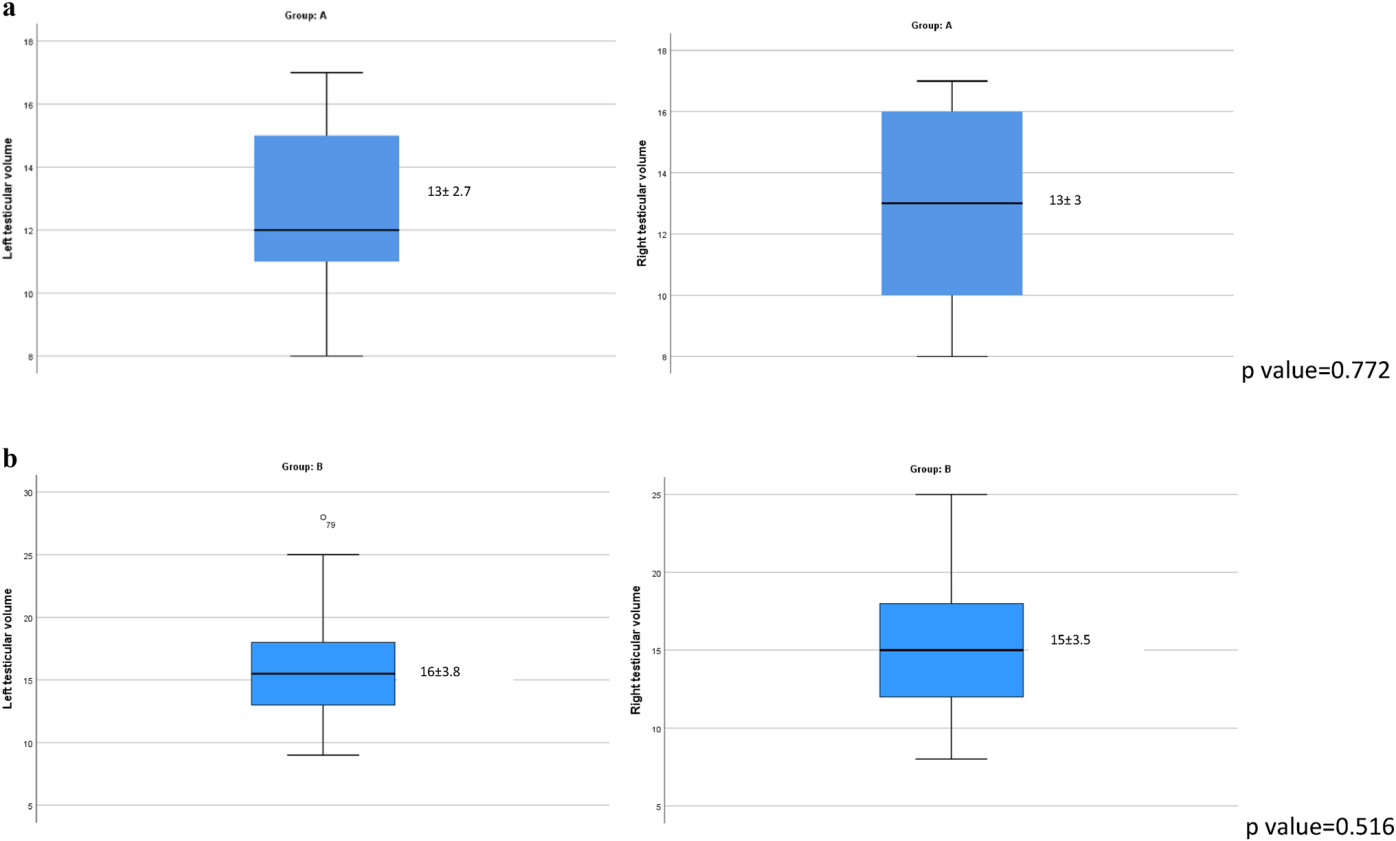
**a** Average testicular volume analysis found in left and right group A. **b** Average testicular volume analysis found in left and right group B

**Fig. 3:**
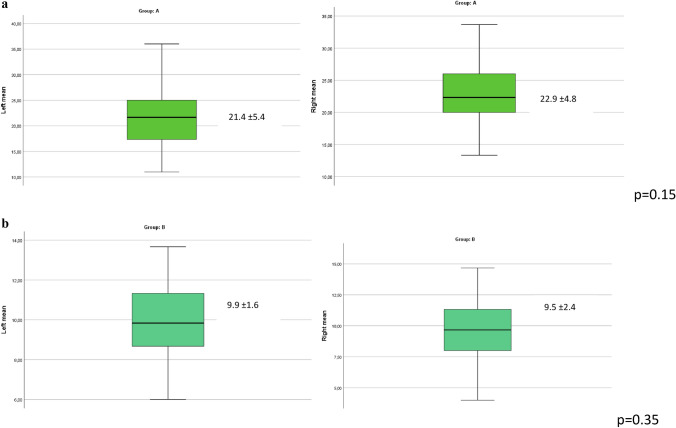
**a** Testicular stiffness value in left and right group A. **b** Testicular stiffness value in left and right group B

Figures 2 and 3 show that, within each group, there were no statistically significant differences in terms of testicular volume and testicular stiffness between the two sides in both groups.

There was a statistically significant difference between the mean testicular volume in participants with semen quality defects and in the control group both in left (*p* < 0.0001) and in right side (*p* = 0.001, Table 3). In fact, group A patients had a testicular volume lower than group B patients.

**Table 3:** Comparison between the average testicular volume in group A and the average testicular volume in group B for both right and left sides

	Left testicular volume[mean (± SD)]	*p* value	Right testicular volume [mean (± SD)]	*p* value
Group A	13 ± 2.7	< 0.0001*	13 ± 3	0.001*
Group B	16 ± 3.8		15 ± 3.5	

*SD* standard deviation

*Statistically significant at *p* < 0.05

In addition, the testicular stiffness value in group A patients was greater than the testicular stiffness value in group B patients in a statistically significant manner (*p* < 0.0001) (Table 4, Fig. 3).

**Table 4:** Comparison between left and right testicular SWE values for both right and left sides

	Left testicular stiffness [mean kPa (± SD)]	*p* value	Right testicular stiffness [mean kPa (± SD)]	*p* value
Group A	21.4 (± 5.4)	< 0.0001*	22.9 (± 4.8)	< 0.0001*
Group B	9.9 (± 1.6)		9.5 (± 2.4)	

*SD* standard deviation

*Statistically significant at *p* < 0.05

**Fig. 4:**
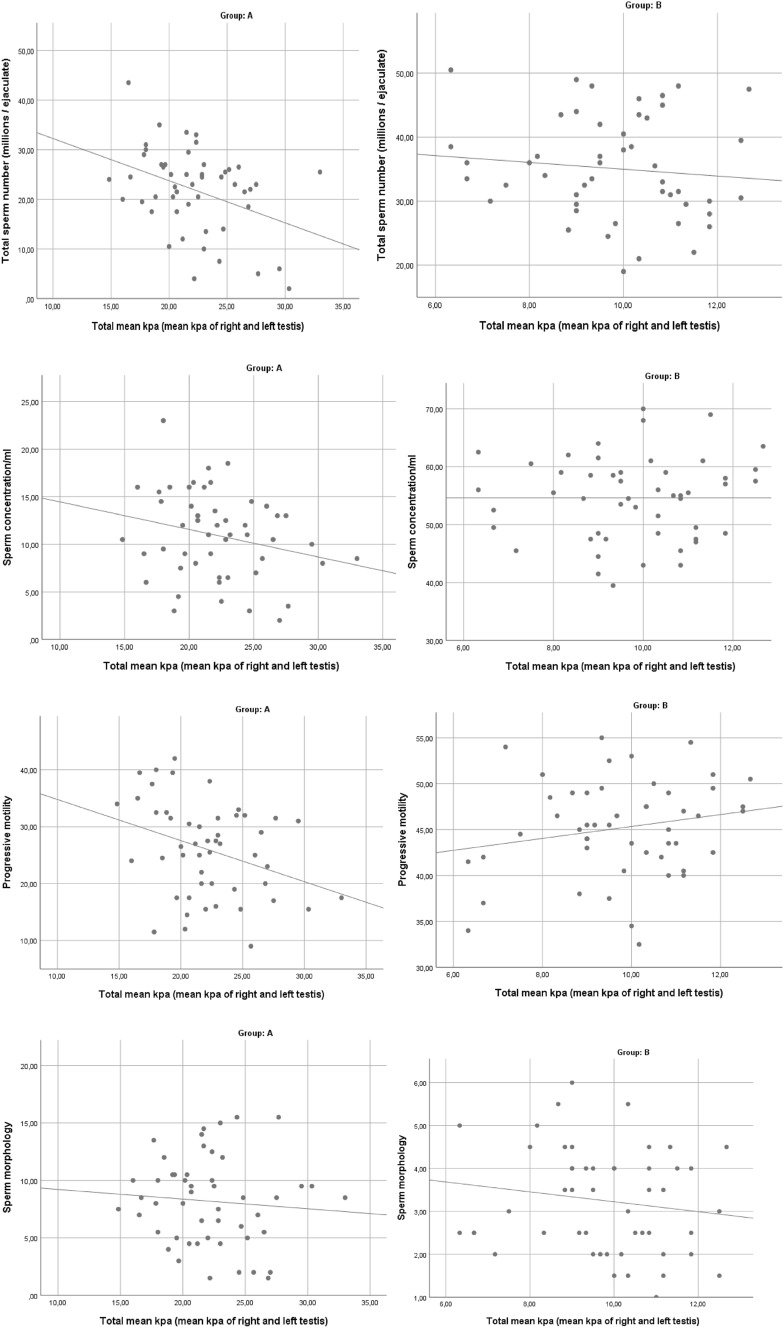
Correlation between testicular stiffness and seminal analysis values of patients in groups A and B

In participants with abnormal semen parameters, there was an inverse correlation between the mean value of testicular stiffness expressed in kPa (left and right testicle) and total sperm count (*p* = 0.005), sperm concentration (*p* = 0.04), and progressive motility (*p* = 0.01) (Table 5, Fig. 4). The correlation was not evident in the group with healthy men (Table 5).

**Table 5:** Correlation between testicular stiffness and spermiogram values of patients in groups A and B

Group	Mean kPa ± SD	*r*	*p* value
A			
Total sperm number (10^6^/ejaculate, mean ± SD)	22.15 ± 3.38	− 0.387	0.005*
Sperm concentration (10^6^/mL, mean ± SD)		− 0.244	0.04*
Progressive motility (PR + NP, mean ± SD)		− 0.336	0.01*
Sperm morphology		− 0.085	0.55
B			
Total sperm number (10^6^/ejaculate, mean ± SD)	9.7 ± 1.6	− 0.10	0.46
Sperm concentration (10^6^/mL, mean ± SD)		− 0.07	0.61
Progressive motility (PR + NP, mean ± SD)		0.19	0.16
Sperm morphology		− 0.15	0.29

*r* Pearson’s correlation

**Statistically significant at *p* < 0.001

*Statistically significant at *p* < 0.10

There are no statistically significant differences between the testicular volume and the elasticity found in group A (left testicular: *p* = 0.240; right testicular: *p* value 0.331).In the same way, there was no statistically significant difference between the testicular volume and the elasticity found by performing SWE to the group B (left testicular: *p *= 0.056; right testicular: *p* value 0.645).

Table 6 showed a statistically significant difference in the mean of the variables relating to the upper right and left poles (*p* < 0.0001), lower right and left poles (*p* < 0.0001), and medial right and left (*p* < 0.0001) in the two subgroups A and B.

**Table 6:** Stiffness value to the upper right and left poles, lower right and left poles, medial right and left region in the two subgroups A and B

Group	*N*	Mean	Std. deviation	*p* value
Left lower pole				
A	50	22.88	11.36	0.0001*
B	50	8.04	3.56	
Left upper pole				
A	50	22.00	7.90	0.0001*
B	50	10.28	2.86	
Left medial				
A	50	21.48	5.45	0.0001*
B	50	9.94	1.61	
Right lower pole				
A	50	24.02	9.07	0.0001*
B	50	10.08	5.47	
Right medial				
A	50	22.91	4.89	0.0001*
B	50	9.59	2.45	
Right lower pole				
A	50	24.02	9.07	0.0001*
B	50	10.08	5.47	

In the Table 7 we analyzed the differences in stiffness between the upper pole, medial region, and lower pole in the same testis in both Group A and Group B; there were no significant differences (*p* > 0.05).

**Table 7:** Differences stiffness in the same testis in regional part (upper. medial and lower) in both group A and group B

	Mean	Std. deviation	*p* value
Group A			
Right upper pole	23.44	8.67	0.756
Right lower pole	24.02	9.07	
Right upper pole	23.44	8.67	0.601
Right medial	22.91	4.89	
Right lower pole	24.02	9.07	0.295
Right medial	22.91	4.89	
Left upper pole	22.00	7.90	0.635
Left lower pole	22.88	11.36	
Left upper pole	22.00	7.90	0.595
Left medial	21.48	5.45	
Left lower pole	22.88	11.36	0.229
Left medial	21.48	5.45	
Group B			
Left upper pole	10.28	2.86	0.082
Left lower pole	8.90	3.56	
Left upper pole	10.28	2.86	0.295
Left medial	9.94	1.61	
Left lower pole	8.90	3.56	0.061
Left medial	9.94	1.61	
Right upper pole	10.52	4.41	0.361
Right lower pole	10.08	5.47	
Right upper pole	10.52	4.41	0.052
Right medial	9.59	2.45	
Right lower pole	10.08	5.47	0.425
Right medial	9.59	2.45	

**Fig. 5:**
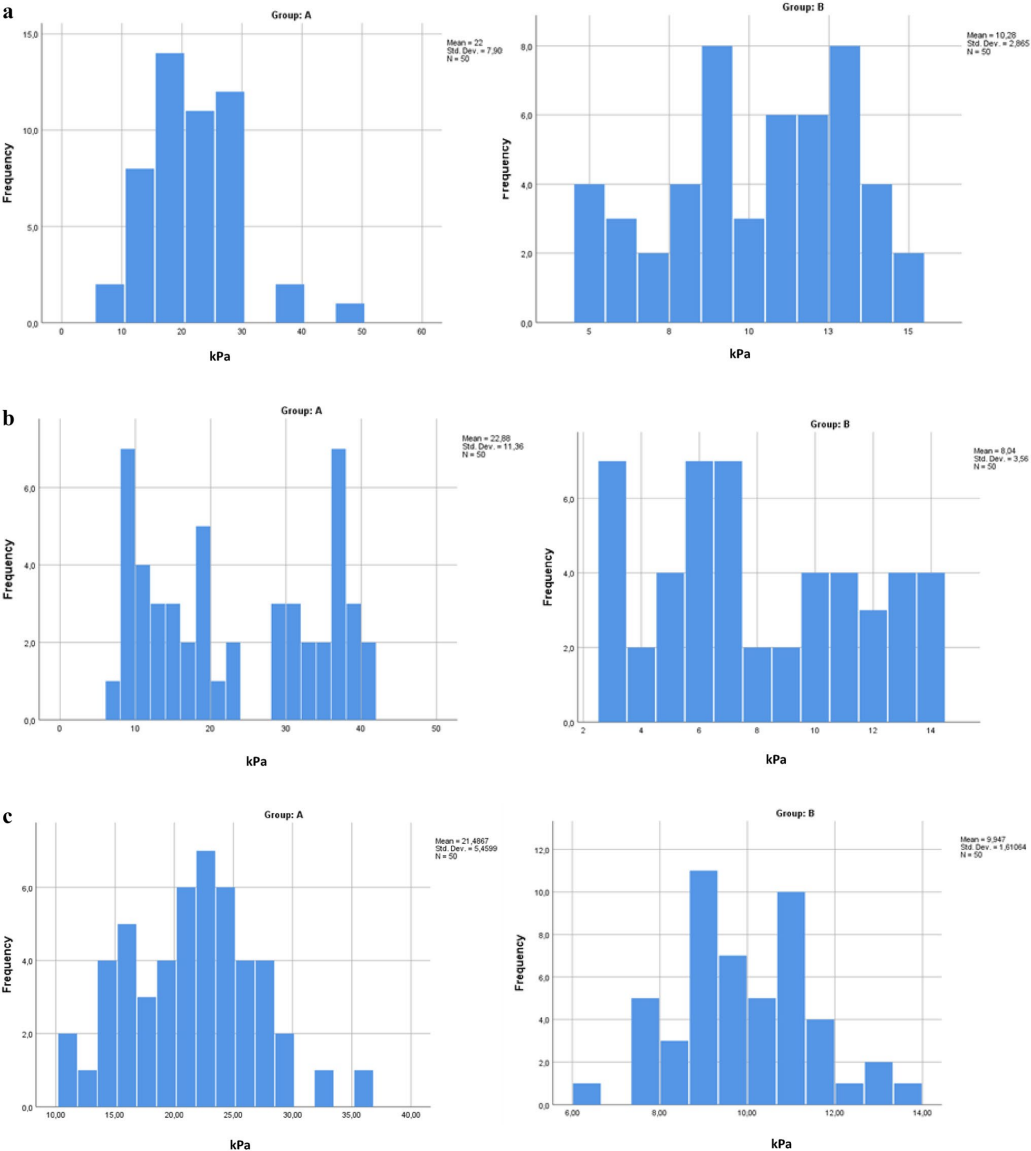
Frequency of left testis stiffness values found in the upper pole (**a**), lower pole (**b**), and medial region (**c**) of both groups A and B

**Fig. 6:**
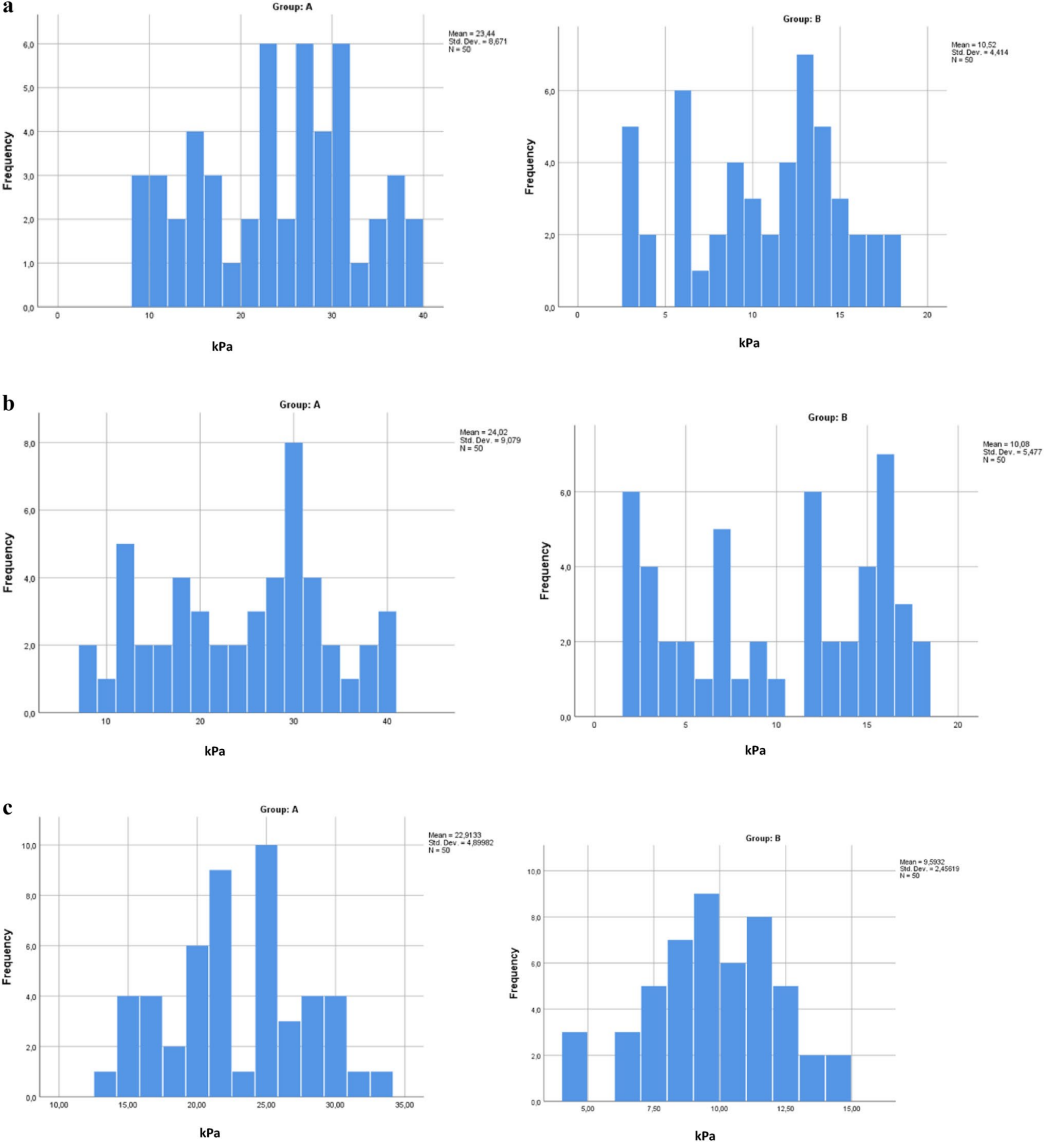
Frequency of right testis stiffness values found in the upper pole (**a**), lower pole (**b**), and medial region (**c**) of both groups A and B

In Figs. 5 and 6, we have reported the frequencies of the stiffness values found in the testicular region (both left and right) in both group A and group B.

## Discussion

Based on the previous literature, approximately 50% of the cases of infertility are caused due to male factors [2]. Oligo-astheno-teratozoospermia syndrome (OAT) is a frequent occurrence in couples with infertility problems. Approximately 30% of all infertile men are unaware of the underlying cause of their infertility. The diagnosis of OAT is performed on the basis of semen analysis results, but the causes of OAT syndrome are complex; in fact there may be genetic, environmental or iatrogenic causes that cause alterations of semen analysis [14].

Testicular biopsy could be considered as the gold standard of investigation to understand the cause of testicular damage, but it is not always feasible due to the invasive nature, associated costs, and possible side effects [12].

If performed well and analyzed properly, the semen analysis can be a good diagnostic aid, along with the andrological examination and bilateral testicular ultrasound, in the diagnostic work-up of seminal problems in males [14].

SWE is an operator-independent method that can be easily reproduced [15, 16]. Moreover, it can be used to obtain a quantitative value of the analyzed tissue and to eventually compare the results obtained following the targeted treatments [17].

In fact, testicular kPa values, which represent parenchymal elasticity, are inversely correlated with sperm values.

Moreover, in our study, the US SWE showed that in Group A, the testicular volume was lower than in the control group (Group B). Previously reported results of ultrasound evaluation of testicular volume were in agreement with ours [18]. This is in accordance with Ehala-Aleksejev et al. who found that the testicular volume measured by US examination correlated negatively with seminal parameters [19]. Erdogan et al. showed that in patients with spermatic problems, testicular SWE value was significantly higher than in patients without spermatic problems, which is in agreement with our observations. It is possible that the parenchymal damage that causes seminal fluid defects simultaneously decreases the elasticity of the testes [20]. Rocher et al. [12], however, did not find a correlation between testicular volume and stiffness value. This difference in findings could be explained by the use of different technique; the previous authors used strain elastography, whereas we performed SWE.

SWE is a non-invasive, inexpensive, and a well-tolerated diagnostic procedure. In fact, SWE could represent a more accurate method to evaluate testicular parenchymal stiffness than the testicular palpation method performed during the physical examination to assess parenchymal elasticity. Zhang et al. demonstrated by SWE that in rabbits with testicular damage (following artificial testicular torsion and subsequently objectified by tissue biopsy) there was an increase in testicular rigidity. In addition, they found that the higher the stiffness values, the worse the spermatogenesis and morphology of the spermatozoa [21].

Findings presented by Yavuz et al. were similar to our results, showing that sperm count is inversely correlated with testicular stiffness value (expressed in kPa), as they found a negative correlation between testicular values and sperm count with SWE. They concluded that testicular damage, on the one hand, decreased sperm count, and on the other hand, increased testicular kPa. Our study made an extra leap since the study by Yavuz et al. did not compare the results with those of a control group and also did not exclude patients potentially affected by testicular pathologies that could generate enrollment bias [11].

Rocher et al. performed a study that identified infertile patients in various groups: OAT syndrome, obstructive azoospermia, Klinefelter syndrome non-obstructive azoospermia (KS–NOA), non-Klinefelter syndrome non-obstructive azoospermia (NOA), and varicocele [12]. They found that KS-NOA patients had higher stiffness values than the NOA group. This is probably because the Leydig cell hyperplasia cluster may cause the formation of micronodules and, therefore, lead to a higher stiffness [22]. In our study, we excluded patients with Klinefelter syndrome to avoid bias related to histological changes in these patients.

Moreover, contrary to the study performed by Rocher et al., we excluded patients diagnosed with varicocele. In fact, this pathology could affect testicular elasticity. Dede et al. demonstrated a decrease in testicular elasticity in patients with varicocele [9].

The strengths of this study were the case–control prospective design and the measurement of the value of the testicular stiffness in three ROIs (1/3 upper pole, 1/3 central zone, 1/3 lower pole) for a greater accuracy of the measurements, since there is no agreement among the studies regarding which part of the testes should be used for the measurement.

In addition, the presence of another urologist experienced in SWE who evaluated the measurements helped ensure that the measurements were accurate.

Limitations of this study included the lack of histopathologic data obtained from testicular biopsies and the small sample size. In addition, hormonal determinations of the participants were not evaluated.

## Conclusions

SWE is an easy, reproducible, operator-independent, non-invasive, and inexpensive technique that provides information about the male gonads and their functionality. It is able to differentiate between the testicles capable of performing spermatogenesis and testicles with spermatic problems. Moreover, with the possibility of obtaining an absolute value, expressed in kPa, it is possible to follow the evolution of OAT syndrome and to compare the values obtained after pharmacologic therapy. SWE is a promising technique to aid in the diagnosis of OAT in male infertile patients.
